# Surgery-Hernia

**Published:** 1904-06-18

**Authors:** 


### SURGERY.—HERNIA.

*(Concluded from page 191.)*

**Intestinal Obstruction due to an Obturator Hernia.**— Lunn[8] relates the case of a woman aged 61, who was admitted to the Marylebone Infirmary. Four days previously she had been taken with acute pain in the abdomen followed by vomiting. The patient was exceedingly obese. On admission the general condition was good, tongue clean, and no distension of the abdomen. She had a right reducible hernia and nothing definite could be made out on the left side. The rectum was emptied by enema. Twenty-four hours after admission she began to vomit some doubtfully fæcal material, her tongue became dry, and pulse rather suggestive of peritonitis. The stomach was washed out and the abdomen opened on the middle line; distended dark small intestines presented covered with lymph. On tracing the distended bowel to the left iliac fossa collapsed intestines were seen apparently bound down to the back of the os pubis, and during the examination fæces welled up from the left iliac fossa. The collapsed intestines were found to be adherent below the horizontal ramus of the os pubis towards the direction of the obturator foramen. On examining the upper part of the left thigh a fulness could be detected below Poupart's ligament on the inner side of the femoral vessels. This swelling was cut down upon and a piece of gangrenous omentum found very adherent to the surrounding tissues. This was ligatured and removed and the ruptured sloughing bowel found to have been strangulated at the entrance to the obturator foramen. The whole lumen of the bowel had ulcerated through except about a quarter of an inch. A resection was performed with Murphy's button, but the patient died next day.

Post-Operative Ventral Hernia.—Carless[9] in a review of the recent literature of hernia gives the views of Hahn and Anderson. They point out the importance of suturing the abdominal wall layer by layer to prevent this hernia, and they both insist that fasciæ and aponeuroses are more resistant than muscle fibres and therefore should be included, whilst the muscular tissue may be left aside. The author is doubtful of the advisability of this; he says the importance of securing fasciæ and aponeuroses cannot be doubted, but if in addition a good hold of divided muscular planes is also secured, there is not much likelihood of a subsequent hernia. Hahn advises catgut or silkworm gut, and keeps the patient recumbent for not less than three weeks. In spite of these precautions hernia is commonly seen especially after draining abscesses. Witzel was the first to suggest embedding a silver-wire filigree in the tissues, and this plan has been followed by success. Bartlett[10] relates seven cases. He points out that scars in the abdominal wall usually spread laterally and not longitudinally to any extent, and therefore it is needless to strengthen the scar with a number of wires which run parallel to the axis of the incision. His filigree therefore consists merely of a suitable number of transverse loops held together by a longitudinal bar in the centre. The loops must be of sufficient length to reach across the gap and extend an inch or two into the tissue-layers where they will be subsequently anchored by newly-formed scar-tissue. The longitudinal strand in the centre corresponds with the length of the incision. The wire netting must be made to suit each particular case and is easily constructed on a board with nails driven in at suitable intervals, the wire being wound round them.

Taxis.—In the same review as above[11] attention is called to a paper by Lanz entitled "Away with Taxis," and the author points out that it is often injudiciously used. Haberer records three instances of grave mischief following forcible taxis; in one a gangrenous hernia had been reduced with a fatal result, in the second the mesentery of the strangulated gut had been torn with hæmorrhage into the sac, recovery following operation, and in the third the mesentery was badly torn, necessitating removal of eighty-three centimetres of the small intestine. As other examples of the harm that may be done, Hilgemunir refers to eight reductions *en masse*, one torn sac and one contused testicle. Lanz observed four cases in one year, one a reduction *en masse*, and three reductions of gangrenous intestine—all of which proved fatal. Taxis should be sparingly employed in cases of acute strangulation, thus in small, painful herniæ, recently developed or if strangulation has followed their first appearance, or when the hernia has been strangulated some time. Taxis can be employed with most advantage where the hernia is of some size, particularly if inguinal, when the symptoms are not severe, and if it has been successful on former occasions. Gray[12] considers that taxis should never be practised in strangulated cases.

[8] Lancet, Nov. 7, 1903. [9] Pract., Feb. 1904. [10] Annals of Surg., July, 1903. [11] Pract., Feb. 1903. [12] Med. Rec., Feb. 6, 1904.

